# Impact of early onset of chronic physical multimorbidities on schizophrenia spectrum disorder treatment outcome

**DOI:** 10.1192/j.eurpsy.2022.324

**Published:** 2022-09-01

**Authors:** I. Šimunović Filipčić, I. Filipcic

**Affiliations:** 1 University Hospital Zagreb, Department Of Psychiatry And Psychological Medicine, Zagreb, Croatia; 2 Psychiatric Clinic “Sveti Ivan”, Psychiatry, Zagreb, Croatia

**Keywords:** Schizophrenia spectrum disorders, multimorbidity, physical comorbidities, rehospitalisation

## Abstract

**Introduction:**

Despite of the heightened risks and burdens of physical comorbidities across the entire schizophrenia spectrum disorders (SSD), relatively little is known about physical multimorbidity (CPM) in this population. The study’s main objective was to explore the differences in the CPM prevalence between SSD patients and the general population (GEP).

**Objectives:**

The primary outcome was to explore the difference in CPM prevalence in the younger SSD and GEP groups (<35 years).The secondary outcome was the number of psychiatric readmissions.

**Methods:**

This nested cross-sectional study enrolled 343 SSD patients and 620 GEP participants.

**Results:**

Younger SSD patients had more than three-fold higher odds for CPM than GEP. We also demonstrated an association between the presence of CPM and the number of psychiatric admissions in the SSD population independently of possible confounders. We did not observe significant interaction of CPM and age in the prediction of clozapine use. Younger women with SSD had statistically significant, almost four-fold higher odds of CPM than women from GEP.

**Conclusions:**

This study suggests that women with SSD are at increased physical comorbidity risk compared to men, particularly early in the course of psychiatric illness. Our results highlight the importance of addressing physical health from the first contact with a mental health service to preserve general health, and provide the best possible treatment outcome. Treatment of SSD must be customized to meet the needs of patients with different physical multimorbidity patterns.

**Disclosure:**

No significant relationships.

